# Molecular mechanisms of PI3Kα activation by small-molecule activator 1938 and cancer-specific mutation H1047R

**DOI:** 10.1038/s41421-025-00833-w

**Published:** 2025-09-19

**Authors:** Xiao Liu, Qingtong Zhou, Yanyan Chen, Wei Han, Jie Li, Yiting Mai, Ming-Wei Wang

**Affiliations:** 1 Department of Pharmacology, School of Basic Medical Sciences, Fudan University, Shanghai, China; 2 Research Center for Medicinal Structural Biology, National Research Center for Translational Medicine at Shanghai, State Key Laboratory of Medical Genomics, Ruijin Hospital, Shanghai Jiao Tong University School of Medicine, Shanghai, China; 3 Research Center for Deepsea Bioresources, Sanya, Hainan China; 4 Engineering Research Center of Tropical Medicine Innovation and Transformation of Ministry of Education, School of Pharmacy, Hainan Medical University, Haikou, China; 5 Department of Chemistry, School of Science, The University of Tokyo, Tokyo, Japan

**Keywords:** Cryoelectron microscopy, Phosphoinositol signalling

Dear Editor,

Phosphoinositide 3-kinases (PI3Ks) play central roles in regulating critical cellular processes such as metabolism, cell survival, and motility, with their dysregulation implicated in cancer, inflammation, and metabolic disorders^1–3^. Of special interest is PI3K alpha (PI3Kα), a heterodimer of p110α and p85α. Cancer-specific mutations in p110α, such as H1047R, result in constitutive PI3Kα signaling, contributing to tumorigenesis by sustaining aberrant AKT-mTOR pathway^4–7^. While PI3K inhibitors have dominated therapeutic strategies for cancer and immune disorders, emerging evidence highlights the benefits of PI3Kα activation in tissue protection and regeneration^8–12^. For example, the small-molecule activator UCL-TRO-1938 (referred to as 1938 hereafter) selectively enhances PI3Kα activity, demonstrating cardioprotection against ischemia-reperfusion injury and promoting nerve regeneration in preclinical models^9^. Intriguingly, 1938 synergizes with H1047R to amplify PI3Kα activity, suggesting distinct yet complementary mechanisms that promote PI3Kα activation^9^. However, structural insights into how 1938 modulates PI3Kα conformations and cooperates with H1047R remain elusive.

Here, we present cryo-electron microscopy (cryo-EM) structures of wild-type (WT) PI3Kα in complex with the activator 1938, the unliganded PI3Kα H1047R mutant, and the PI3Kα H1047R mutant in complex with 1938 at resolutions of 3.17 Å, 3.09 Å, and 2.94 Å, respectively (Fig. 1; Supplementary Figs. S1, S2, and Table S1). Our comparative analysis reveals distinct conformational changes induced by 1938 and H1047R, with similar conformational changes in the ABD and iSH2 domains that relieve p85α autoinhibition on p110α.

**Fig. 1:**
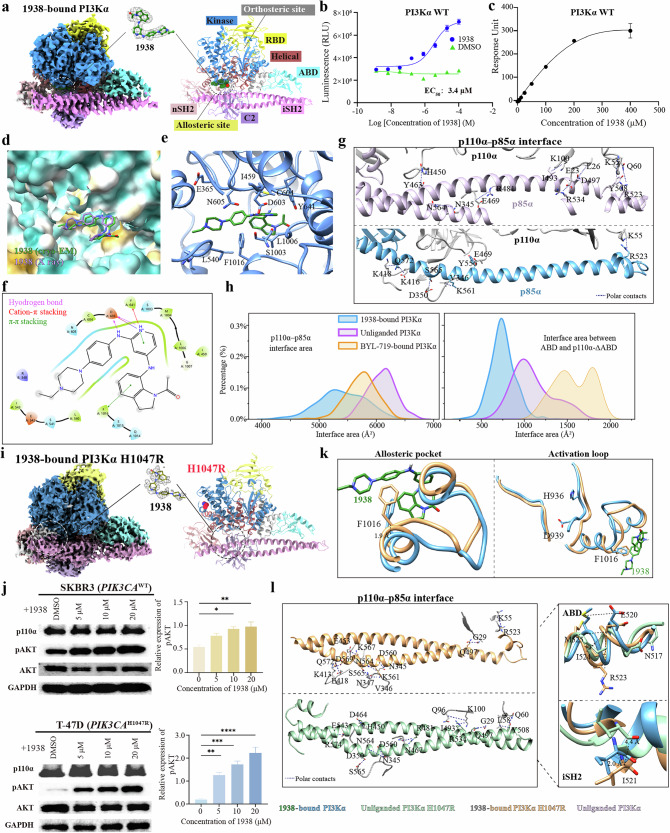
Structural insights into PI3Kα activation by small-molecule activator 1938 and cancer-specific mutation H1047R. **a** Cryo-EM density map and corresponding atomic model for PI3Kα in complex with 1938 (highlighted in green). **b** Compound 1938 dose-dependently activates wild-type (WT) PI3Kα with an EC_50_ value of 3.4 μM. **c** Representative SPR sensorgram of 1938 binding to PI3Kα WT with a *K*_D_ value of 253 ± 69 μM. **d** A surface representation comparing the binding pose of 1938 in cryo-EM (green) and X-ray crystallography (purple) within PI3Kα. **e**, **f** Structural (**e**) and schematic (**f**) representation of the interactions between 1938 and PI3Kα in the cryo-EM structure. Residues involved in 1938 recognition are shown as sticks. **g** Interaction network at the interface among the iSH2, ABD, and C2 domains is disrupted in the 1938-bound structure (bottom) compared to the unliganded PI3Kα (PDB ID: 7MYN, top). **h** Distribution of the interface area between p110α and p85α (left), and between ABD and the p110α without ABD (right), from the last 500 ns of MD simulation trajectories, calculated by FreeSASA 2.0. **i** Cryo-EM density map and corresponding atomic model for the 1938-bound H1047R complex, shown in two orientations. The EM density of 1938 is shown as a mesh. **j** Compound 1938 activates PI3Kα/AKT signaling in breast cancer cells. SKBR3 (*PIK3CA*^WT^) and T-47D (*PIK3CA*^H1047R^) cells were treated with the indicated concentrations of 1938 for 5 min at 37 °C. Western blot analysis shows dose-dependent upregulation of pAKT (S473) following 1938 treatment (left). GAPDH: loading control. Bar graphs show densitometric analysis of pAKT (S473) and GAPDH (mean ± SEM, *n* = 3) (right). **k** Conformational comparison of F1016 (left) and the activation loop (right) between 1938-bound H1047R and 1938-bound WT PI3Kα structures. **l** The interaction network at the p110α–p85α interface is further disrupted in the 1938-bound H1047R structure (top) compared to the unliganded H1047R (bottom).

The cryo-EM structure of the 1938-bound PI3Kα complex closely aligns with the X-ray structure of 1938-bound p110α (PDB code: 8OW2, residues 105‒1048 of p110α, without the regulatory subunit p85α)^9,13^, with a Cα root-mean-square deviation (RMSD) of 1.05 Å (Fig. 1d; Supplementary Fig. S3b). These structures reveal that 1938 occupies an allosteric pocket at the interfaces among the C2, helical, and kinase domains, obviously different from the orthosteric inhibitor BYL-719 (Fig. 1a). 1938 adopts a “V”-shaped conformation, with the acetylated indoline moiety nestled within a sub-pocket delineated by I459, L1006, and F1016, where it engages in a π‒π interaction with F1016 (Fig. 1d, e). The other segment of 1938 aligns in a linear conformation, with the core pyridine ring forming a π‒π stacking interaction with Y641. Meanwhile, the nitrogen within the pyridine is positively charged, making salt bridges with the sidechain of D603, while the latter also forms one hydrogen bond with the nitrogen atom connecting the pyridine and benzene rings. Additionally, the central benzene is clasped by N605 and F1016, allowing the linked piperazine ring to approach the helical domain, where it makes massive hydrophobic contacts with P539, L540, and L570 (Fig. 1e, f). This observation was further supported by molecular dynamics (MD) simulations (Supplementary Fig. S3c, d).

Binding of 1938 to WT PI3Kα induces conformational changes compared to the unliganded and BYL-719-bound PI3Kα (PDB IDs: 7MYN and 7MYO), with Cα RMSD values of 1.35 Å and 1.37 Å, respectively (Supplementary Fig. S3e). The ABD of the 1938-bound PI3Kα undergoes an outward displacement of 3.1 Å compared to its unliganded state, and a larger displacement of 3.5 Å compared to the BYL-719-bound state (Supplementary Fig. S3f). This is due to the rotation of the sidechain of E85. Consequently, the enhanced flexibility of the ABD allows for large fluctuations in the iSH2 junction upon 1938 binding (Supplementary Fig. S3f). These movements reorganize the interface among iSH2, ABD, and C2 domains (Fig. 1g), leading to a weakened interaction network between p110α and p85α. Consistently, in our MD simulation, the p110α–p85α interface area in the 1938-bound PI3Kα (5400 Å^2^) is significantly smaller than that observed in the unliganded (6092 Å^2^) or BYL-719-bound (5742 Å^2^) states (Fig. 1h). Furthermore, 1938 enhances the kinase activity of a truncated PI3Kα complex comprising p110α and the niSH2 domains of p85α (lacking nSH3, BH, and cSH2 domains), although with reduced potency (EC_50_ = 61.7 μM vs 3.4 μM for full-length WT PI3Kα) (Fig. 1b; Supplementary Fig. S4).

Looking at the other regions of p110α, residues 1002–1016 shift from the helical domain towards the active site, opening the allosteric site to accommodate 1938 binding (Supplementary Fig. S3g). The 1938-bound structure also reveals conformational changes in the activation loop (residues 933–958) and P loop (residues 771–777) compared to the unliganded state (Supplementary Fig. S3h). Additionally, a 3.8 Å lateral displacement in the membrane-binding loop (residues 966–974) was observed in the Cα position of C971 (Supplementary Fig. S3i). These changes illustrate the structural impact of 1938 on PI3Kα.

Compared to the unliganded WT PI3Kα, the overall structure of the unliganded PI3Kα 1047R is globally similar, with a Cα RMSD of 0.87 Å. However, notable differences were observed at the mutation site (H/R1047), the membrane-binding loops, and ABD (Supplementary Fig. S5). The sidechain of R1047 rotates toward kα11, forming hydrogen bonds with T972 and N1044, and π-stacking interactions with F977, which are absent in the WT structure (Supplementary Fig. S5b). The density for the p110α tail segment (residues 1050–1062) was missing, indicating its highly dynamic and disordered nature. The membrane-binding loops, including loop 1 (residues 721–727), loop 3 (residues 966–974), and loops in the C2 domain (residues 410–418 and 343–351), underwent varying degrees of displacement (Supplementary Fig. S5f)^14^. At the p110α−p85α interface, the ABD of p110α rotated, resulting in a 1.8 Å Cα shift at residue K55. Meanwhile, the iSH2 domain revolved around T500, inducing a 1.7 Å Cα shift at residue S504 (Supplementary Fig. S5e). These structural changes, distant from the mutation site, suggest that the H1047R mutation impacts not only the kinase domain but also other regions interacting with p85α and the membrane (Supplementary Fig. S5).

The unliganded and BYL-719-bound PI3Kα H1047R complex structures (PDB ID: 8GUB) align well, with a Cα RMSD of 0.60 Å. More significant differences were observed in the iSH2 and kinase domains, with RMSD values of 0.71 Å and 0.92 Å, respectively (Supplementary Fig. S5a). The sidechain conformation of R1047 remains consistent between the two structures, but the hydrogen bond between R1047 and H1048 observed in the BYL-719-bound PI3Kα H1047R structure was broken when unliganded, due to the inward rotation of H1048^15^ (Supplementary Fig. S5b). Additionally, the activation loop was partially disordered, especially in residues 945–950, indicating increased conformational flexibility. Despite these changes, the ATP-binding pocket retained a highly conserved spatial arrangement in both structures (Supplementary Fig. S5c), with only slight movement of the P loop. Furthermore, the membrane-interaction regions — membrane-binding loops 1 and 3, and the P loop, differ from those in the BYL-719-bound PI3Kα H1047R and unliganded WT PI3Kα (Supplementary Fig. S5d, f).

Compound 1938 dose-dependently increased the kinase activity of the PI3Kα H1047R mutant with an EC_50_ value of 22.4 μM (Supplementary Fig. S6a). Consistently, 5-min treatment with 1938 in T-47D cells (*PIK3CA*^H1047R^) induced a dose-dependent increase in pAKT (S473) levels without altering total AKT expression (Fig. 1j). Furthermore, surface plasmon resonance (SPR) analysis validated that 1938 has comparable binding affinities to both WT PI3Kα (*K*_D_ = 253 ± 69 μM) and PI3Kα H1047R (261 ± 45 μM) (Fig. 1c; Supplementary Fig. S6b). These data demonstrate that 1938 functionally synergizes with the PI3Kα H1047R oncogenic mutation to amplify PI3Kα signaling output, possibly uncoupling from the ligand binding kinetics. The 1938-bound PI3Kα H1047R structure more closely resembled that of the unliganded PI3Kα H1047R, with a Cα RMSD of 0.60 Å, lower than that for the 1938-bound PI3Kα WT (0.99 Å) (Supplementary Fig. S6c). This suggests that the addition of 1938 induces moderate conformational changes, particularly within the allosteric binding pocket (Supplementary Fig. S6c). Different from the unliganded PI3Kα, the sidechain of R1047 in the 1938-bound PI3Kα H1047R structure aligned with that observed in the unliganded PI3Kα H1047R, interacting with T972, Q981, N1044, and F977 (Supplementary Fig. S6g). The mutation‑initiated rearrangement at position 1047 is accompanied by increased mobility of kα11 and a slight inward shift of kα10 (residues 1016–1025) and the adjacent loop (residues 1005–1016) in the 1938‑bound PI3Kα H1047R complex compared to the 1938‑bound PI3Kα WT (Fig. 1k). Concurrently, F1016 moves 1.90 Å (Cα) closer to the allosteric binding pocket of 1938, which may underlie the comparatively weak cryo‑EM density for 1938 (Fig. 1k). In addition, the activation loop exhibits subtle repositioning: D933 (DFG motif) shifts 1.0 Å (Cα) toward the ATP site, and K942, a residue critical for substrate engagement, displaces by 3.0 Å (Cα) (Supplementary Fig. S6f). These observations present an allosteric connection between the binding of 1938 and the PI3Kα H1047R mutation, driven by the structural alterations.

The synergistic effect of 1938 and PI3Kα H1047R also extends to other regions of PI3Kα, especially in the P loop of p110α and the iSH2 domain of p85α (Supplementary Fig. S4d). To quantify the closure of the P loop and activation loop, we calculated the minimum distance between the Cα atom of A775 and the sidechain oxygen atoms of D933. Notably, the shortest distances were observed in the BYL-719-bound WT PI3Kα (5.7 Å) and the BYL-719-bound PI3Kα H1047R (8.1 Å), consistent with the presence of the inhibitor. This is followed by the unliganded WT PI3Kα (9.2 Å) and 1938-bound WT PI3Kα (9.9 Å), while both the unliganded PI3Kα H1047R (11.0 Å) and 1938-bound PI3Kα H1047R (11.7 Å) showed significantly larger distances, indicating a more open conformation favoring enzymatic activity (Supplementary Fig. S4d). Looking at the p110α−p85α interface, the iSH2 domain shifted by 2.0 Å at the Cα atom of I521 in comparison to the 1938-bound WT, and by 4.4 Å compared to PI3Kα H1047R (Fig. 1l). This suggests that the 1938‒PI3Kα H1047R combination further disrupts the inhibitory contacts between p85α and p110α to enhance the kinase activity (Fig. 1l; Supplementary Fig. S6e). In agreement with this, our MD simulations showed that the p110α−p85α interface area for the 1938-bound PI3Kα H1047R is the smallest (5283 Å^2^), compared to the 1938-bound WT (5400 Å^2^) and unliganded PI3Kα H1047R (6315 Å^2^), reinforcing the synergistic effect of 1938 and H1047R in reducing the autoinhibition of p85α on p110α (Supplementary Fig. S6d).

Our work establishes a structural paradigm for PI3Kα regulation, where p110α activation and the release of p85α autoinhibition are achieved through multiple yet complementary mechanisms. The discovery of the allosteric site in 1938 opened avenues for designing better agents to treat various diseases. Conversely, the 1938‒H1047R interface could inspire dual-targeting inhibitors to counter oncogenic hyperactivation. Future studies are warranted to elucidate dynamic features beyond current technical constraints of cryo-EM and molecular dynamics approaches.

## Supplementary information

Supplementary information

## Data Availability

All relevant data are available from the authors and/or included in the manuscript or Supplementary Information. The cryo-EM density maps have been deposited in the Electron Microscopy Data Bank (EMDB) under accession codes EMD-63456 (1938-bound PI3Kα complex), EMD-63457 (unliganded PI3Kα H1047R complex), and EMD-63458 (1938-bound PI3Kα H1047R complex). Coordinates have been deposited in the Protein Data Bank (PDB) under accession codes 9LWQ (1938-bound WT PI3Kα complex), 9LWR (unliganded PI3Kα H1047R complex), and 9LWS (1938-bound PI3Kα H1047R complex).
