# Supplementary material for: Molecular mechanisms of PI3Kα activation by small-molecule activator 1938 and cancer-specific mutation H1047R
Source: Cell Discov. 2025 Sep 19;11:77. doi: 10.1038/s41421-025-00833-w (PMC12446426; doi:10.1038/s41421-025-00833-w)
Supplement: Supplementary file 1 — Supplementary information [file 41421_2025_833_MOESM1_ESM.pdf]

Supplementary Information

Materials and Methods

Cryo-EM structures in three distinct states

To elucidate the binding mode of 1938, we determined the 1938-bound PI3Kα wild-type (WT) complex structure. The Bac-to-Bac expression system was used to prepare baculovirus carrying p85α and p110α (**Supplementary Fig. S1a**), followed by infecting High Five™ insect cells for protein expression. 1938 was added to the expressing system at a final concentration of 5 μM, ensuring its incorporation into the PI3Kα complex. A 6×His tag was included at the N terminus of p110α to facilitate purification. The PI3Kα complex was purified as previously described[1-4], and its purity was confirmed by sodium dodecyl sulfate-polyacrylamide gel electrophoresis (SDS-PAGE) (**Supplementary Fig. S1a**). The 1938-bound PI3Kα complex was prepared by incubating the PI3Kα complex (1 mg/mL) with 1938 at a final concentration of 500 μM at 4°C overnight. The homogeneity and dispersion of the particles were determined by negative staining (**Supplementary Fig. S1b**). The vitrified 1938-bound PI3Kα complexes were imaged using a Titan Krios equipped with a Gatan K3 Summit direct electron detector (**Supplementary Table S1**). After cryo-EM data analysis, we obtained the cryo-EM map of the 1938-bound PI3Kα complex at 3.17 Å resolution (**Fig. 1a; Supplementary Fig. S1, and Supplementary Table S1**). Local resolution assessment revealed high-resolution features in the catalytic core of p110α and relatively poor resolution in the flexible regions like ABD (**Supplementary Fig. S1**). The map allowed us to build an unambiguous models including all domains of p110α and the nSH2/iSH2 domains of p85α, and the bound ligand 1938 (**Figs. 1a**). Due to the inherent flexibilities, the SH3, BH, and cSH2 domains of p85α were not well resolved, consistent with previous cryo-EM studies of PI3Kα complex[2, 4].

The above approach was also applied to determine the cryo-EM structures of the unliganded PI3Kα H1047R (**Supplementary Fig. S2**) and the 1938-bound PI3Kα H1047R complex (**Fig. 1i-l; Supplementary Fig. S2f-j**). Notably, without the presence of inhibitors such as BYL-719, PI3Kα H1047R particles tended to aggregate, leading to lower yields. Therefore, increasing the virus load during expression was helpful to complex production. Data collection parameters adhered to the same standards as those for 1938-bound PI3Kα samples, resulting in a cryo-EM map with a resolution of 3.09 Å (**Supplementary Fig. S2**). The resulting model of the unliganded PI3Kα H1047R disclosed a dual-component architecture encompassing all p110α domains and two p85α domains (nSH2 and iSH2) (**Fig. 1i**). Initial structural studies of the 1938-bound PI3Kα H1047R complex were carried out with 1938 at a concentration of 250 nM, which was also introduced during the expression. Through data collection and analysis, we obtained a clear electron density map with a resolution of 2.94 Å (**Supplementary Fig. S2j**). However, 1938 could not be fully modelled due to insufficient electron density, reflecting the increased dynamics when combined with PI3Kα H1047R (**Fig. 1i**).

Expression and purification of the 1938-bound PI3Kα

The pFastBac dual vector (Invitrogen) was used to clone the p110α and p85α, and a 6×His tag was also added for

purification later. Baculoviruses containing the above construct were prepared by the Bac-to-Bac system (Invitrogen) and then used to infect *Trichoplusia ni* High Five™ insect cells. The cells were co-infected with the baculovirus at a cell density of 2.0×10^6 cells/mL. 1938 was added to the system at a concentration of 5 μM. Forty-eight hours later, the infected cells were harvested and stored at −80 °C until use.

The expression and purification methods were similar to that described previously[1-4]. For the purification of the 1938-bound WT complex, cell pellets were thawed and resuspended in buffer A (20 mM Tris [pH 8.0], 100 mM NaCl, 5% [vol/vol] glycerol, 20 mM imidazole, and 0.5% [vol/vol] Triton X-100, and 2 mM β-mercaptoethanol) supplemented with protease inhibitor cocktail (TargetMol). Cells were lysed by dounce homogenization (Sigma-Aldrich) followed by centrifugation for 1 h at 140,000 × *g* at 4°C. The supernatant was then passed through a 0.45 μm cellulose acetate membrane filter (Merck Millipore) and incubated with nickel-nitrilotriacetic acid resin (Cytiva) for 2 h at 4°C. The resin was thoroughly washed with buffer B (20 mM Tris-HCl [pH 8.0], 150 mM NaCl, 20 mM imidazole, 5% [vol/vol] glycerol, and 2 mM β-mercaptoethanol) and eluted with buffer C (20 mM Tris-HCl [pH 8.0], 100 mM NaCl, 300 mM imidazole, 5% [vol/vol] glycerol, and 2 mM β-mercaptoethanol). The eluate was concentrated to 500 μL using an Amicon 50-kDa centrifugal filter (Millipore) and injected into a Superdex 200 Increase 10/300 GL gel filtration column (Cytiva) pre-equilibrated with buffer D (20 mM HEPES [pH 7.6], 100 mM NaCl, and 5 mM DTT). The fractions were identified by SDS-PAGE and concentrated to 1.0 mg/mL. The complex was incubated with 1938 at the final concentration of 500 μM overnight at 4°C for cryo-EM examination.

Expression and purification unliganded and 1938-bound PI3Kα H1047R

The expression and purification methods were the same as described above[2]. During expression, the PI3Kα inhibitor BYL-719 was not present. To enhance the yield, an increased virus load was introduced. During purification, optimal experimental conditions were applied to avoid protein aggregation. For the 1938-bound PI3Kα H1047R complex, 250 nM of 1938 was added during expression.

Cryo-EM data collection

Cryo-EM grids were prepared with the Vitrobot Mark IV plunger (FEI) set to 4°C and 100% humidity. Three-microliters of sample solution was applied to freshly glow-discharged cuprum R1.2/1.3 holey carbon grids (Quantifoil), allowed to incubate for 5 s, and then blotted for 4 s on both sides with a blot force of 1. After that, the grids were flash-frozen in liquid ethane for vitrification.

Data acquisition was performed on a Titan Krios microscope (ThermoFisher Scientific), operating at 300 kV and equipped with a Gatan K3 direct electron detector. A nominal magnification of ×46,685 was used in counting mode with serial EM3.7 (ThermoFisher Scientific), corresponding to a calibrated pixel size of 1.071 Å. Images were obtained with a defocus span from −1.5 to −2.5 μm. An accumulated dose of 70 electrons per $Å^2$ was fractionated into a movie stack of 36 frames.

Image processing

MotionCor2.1 was performed to the frame-based motion-correction algorithm to generate dose-fractionated image stacks[5]. A sum of all frames, filtered according to the exposure dose, in each image stack was used for further processing. Contrast transfer function (CTF) parameters for each micrograph were determined by Gctf v1.06[6]. Subsequent analysis steps, encompassing particle picking, extraction, two-dimensional (2D) classification, three-dimensional (3D) classification, *ab-initio* reconstruction, and local refinement, were executed using CryoSPARC v4.

For the dataset of the 1938-bound WT complex, the previously resolved structure of PI3Kα WT (PDB ID: 7MYN) was used as a reference for automatic particle picking. Particle picking and extraction yielded 6,924,249 particles, which were subjected to several rounds of 2D classification, producing 545,384 particles with well-defined averages. This subset of particle projections was subjected to *ab-initio* reconstruction, resulting in one well- defined subset that reconstructed as one reference model. Further 3D classification focusing on the alignment of the whole particles by using the above reference model produced one high-quality subset of 226,201 particles. The final structure, achieved through local refinement, CTF refinement, and Bayesian polishing reached global resolutions of 3.17 Å at a Fourier shell correlation (FSC) of 0.143.

For the dataset of the unliganded PI3Kα H1047R complex, the data collection process yielded a total of 6,339 micrographs. After subjecting the particles to preprocessing, template-based selection, and extraction, we obtained a dataset of 5,286,255 particle images. These particles were then subjected to 2D classification, which allowed us to differentiate between high-quality classes with well-defined features and lower-quality classes. The high-quality particles from these classifications were merged and used for *ab-initio* reconstruction, generating initial templates that served as the foundation for further analysis. Employing these templates, we conducted 3D classification on a subset of 1,851,953 particles to achieve an enhanced selection of high-quality particles. To prepare for the final refinement step, the selected high-quality particles were combined and underwent low pass filtering at a resolution of 20 and 25 Å. Finally, 440,046 of these refined particles were used in the final refinement process, leading to the generation of a density map with a resolution of 3.09 Å at a FSC of 0.143.

For the dataset of the 1938-bound PI3Kα H1047R complex, we obtained a total of 7,768,343 particle images after completing preprocessing and particle picking. Following 2D classification of all particles, we selected 480,707 well-defined particles for 3D classification to produce high-quality classes. This process yielded 238,454 particles that were used for an initial round of refinement, resulting in a density map that served as a template. Utilizing this refined template, we conducted a new round of 3D classification on all original particles. The high-quality classifications from this step were combined, totalling 768,343 particles. These particles then underwent another round of 3D classification yielding 2,446,767 high-quality particles. Using these particles, we generated a new template through *ab-initio* reconstruction, followed by a final refinement process. Ultimately, this led to 1,148,844 particles contributing to the construction of a

density map with a resolution of 2.94 Å at a FSC of 0.143.

Model building and refinement

Structures of WT derived from PDB entry 7MYN was rigid body fitted to the density. Ligand coordinates and geometry restraints were generated using electronic Ligand Builder and Optimization Workbench (eLBOW)[7] and fitted to the cryo-EM density by LigandFit GUI[8] in PHENIX (v1.18.2- 3874). All models were fitted to the EM density map using UCSF Chimera followed by iterative rounds of manual adjustment and automated rebuilding in COOT and PHENIX, respectively. The final model statistics were validated using comprehensive validation (cryo-EM) in PHENIX and provided in the **Supplementary Table S1**. All structural figures were prepared using Chimera[9], Chimera X[10], and PyMOL (Schrödinger, LLC.).

Molecular dynamics (MD) simulation

It was performed with Gromacs 2021.4[11]. The MD simulation of the 1938-bound PI3Kα was built based on the cryo-EM structure and prepared by the Protein Preparation Wizard (Schrödinger 2023-2)[12] to add missing atoms. The protein chain termini were capped with acetyl and methylamide. All titratable residues were left in their dominant state at pH 7.0. To build MD simulation systems, the complexes were solvated with 0.15 M NaCl in explicit TIP3P waters using CHARMM-GUI Solution Builder (https://www.charmm-gui.org/?doc=input/solution)[13]. The CHARMM36m force field[14] implanted in CHARMM-GUI webserver was adopted for protein and salt ions. The parameter of 1938 was generated using the CHARMM General Force Field (CGenFF) webserver (https://cgenff.com/)[15]. The Particle Mesh Ewald (PME) method was employed to treat all electrostatic interactions beyond a cut-off of 10 Å and the bonds involving hydrogen atoms were constrained using LINCS algorithm[16]. The complex system was first relaxed using the steepest descent energy minimization, followed by slow heating of the system to 310 K with restraints. The restraints were reduced gradually over 10 ns. Finally, restrain-free production run was carried out for each simulation, with a time step of 2 fs in the NPT ensemble at 310.15 K and 1.0 bar using the V-rescale thermostat and the isotropic Parrinello-Rahman barostat[17], respectively. The interface area was calculated by the program FreeSASA 2.0, using the Sharke-Rupley algorithm with a probe radius of 1.2 Å[18]. Similar simulation procedure and analysis were adopted for the MD simulations of the unliganded PI3Kα, BYL-719-bound PI3Kα, unliganded PI3Kα H1047R and 1938-bound PI3Kα H1047R complexes. The computational scripts and parameters files for the MD simulations were freely available on GitHub (https://github.com/RuijinHospitalRCMSB/MD_PI3K).

Surface plasmon resonance

Surface plasmon resonance (SPR) analysis was performed on a Biacore 8K instrument (Cytiva) using CM7 sensor chips at room temperature (RT), as previously described. WT PI3Kα and its mutant (1 mg/mL stock) were diluted to 20 μg/mL in acetate buffer (pH 4.5) and immobilized onto the chip surface via a standard amine-coupling protocol using an amine coupling kit (Cytiva). To determine the optimal kinetic model, compound 1938 was serially diluted in DMSO at a 1:2

ratio, with the highest concentration set at 400 μM. Analyte solutions were flowed over the chip surface at 30 μL/min through flow cell 1 (fc1, reference surface without immobilized protein) and flow cell 2 (fc2, protein-coupled surface). Each cycle comprised a 60-second association phase followed by a 60-second dissociation phase. The running buffer was PBS-T supplemented with 5% DMSO, and a solvent correction procedure was applied. Sensorgrams were double-referenced by subtracting signals from the reference flow cell (fc1) and buffer injections. Binding affinity (K_D) was analyzed using Biacore Insight Evaluation software (v4.0.8.20368), with steady-state affinity derived from fitting the binding curves. All measurements were performed in triplicate.

Kinase activity

Kinase activity was measured using the ADP-Glo™ Kinase Assay (Promega). To generate dose-response curves of compound 1938 against PI3Kα WT and H1047R mutant, compound 1938 was serially diluted (1:2 dilution ratio) in DMSO. Reactions were performed in white 384-well plates (Corning) at RT as follows: 1 μL of compound dilution was mixed with 2 μL of enzyme solution (200 nM for PI3Kα WT and truncated p85α, 100 nM for mutant PI3Kα H1047R) and incubated for 10 min. Then, 2 μL of substrate mix (final concentration: 10 μM PIP2 and 400 μM ATP, Merck) was added, followed by 30 min reaction. ADP-Glo reagent was added and incubated for 60 min, after which 10 μL of detection reagent was added and maintained dark for 30 min. Signals were measured using an Envision plate reader (1 s integration time per well). Experiments included three independent replicates.

Evaluation of compound 1938 against PI3Kα mutants was performed identically to the above protocol. Each mutant kinase was adjusted to 40 nM and incubated with 10 μM compound 1938 for 10 min at RT. Subsequently, a substrate mixture (final concentration: 10 μM PIP2 and 400 μM ATP) was added, followed by 60 min reaction. The ADP-Glo reagent was then added and incubated for 60 min, after which the detection reagent was added and protected from light for 30 min prior to signal detection on an Envision plate reader. All experiments were independently repeated three times, and data were analyzed with GraphPad Prism 8.

Western blot

T-47D, MCF-7 and HEK293 cells were cultured in DMEM (ThermoFisher) supplemented with 10% FBS (ThermoFisher), while SKBR3 cells were maintained in McCoy's 5A medium (ThermoFisher) containing 10% FBS. All cell lines were incubated at 37°C under 5% CO_2.

For PI3Kα WT, H1047R and E545K signaling analysis, T-47D and MCF-7 cells were seeded at 300,000 cells/well and SKBR3 at 200,000 cells/well in 12-well plates, followed by overnight adhesion. Cells were then treated with compound 1938 (0, 5, 10, or 20 μM; DMSO ≤ 0.1%) for 5 min at 37°C. For other mutant studies, HEK293 cells were transfected with mutant p110α plasmids using Lipofectamine 3000 (ThermoFisher) for 24 h, with equal amounts of p85α plasmid co-transfected to stabilize p110α expression. Transfected cells were subsequently treated with compound 1938 as above.

After compound treatment, all cells were washed with PBS and lysed in RIPA buffer (Yeasen) containing protease inhibitor cocktail (TargetMol) and 1 mM PMSF (Beyotime). Lysates were centrifuged at 12,000 rpm for 10 min at 4°C, and supernatants stored at −80°C. Protein concentrations were quantified by BCA assay (Beyotime), and 30 μg of protein per sample was separated by SDS-PAGE. Proteins were transferred to 0.45-μm PVDF membranes (Merck), blocked with 5% BSA, and probed overnight at 4°C with primary antibodies: GAPDH (Cell Signaling Technology), p-AKT (Cell Signaling Technology), AKT (Cell Signaling Technology), and p110α (Abclonal). After three 30-min TBST washes, membranes were incubated with HRP-conjugated secondary antibodies (Abcam) for 30 min at RT. Protein bands were visualized by chemiluminescence. Experiments included three independent replicates and presented with ImageJ (1.54 g).

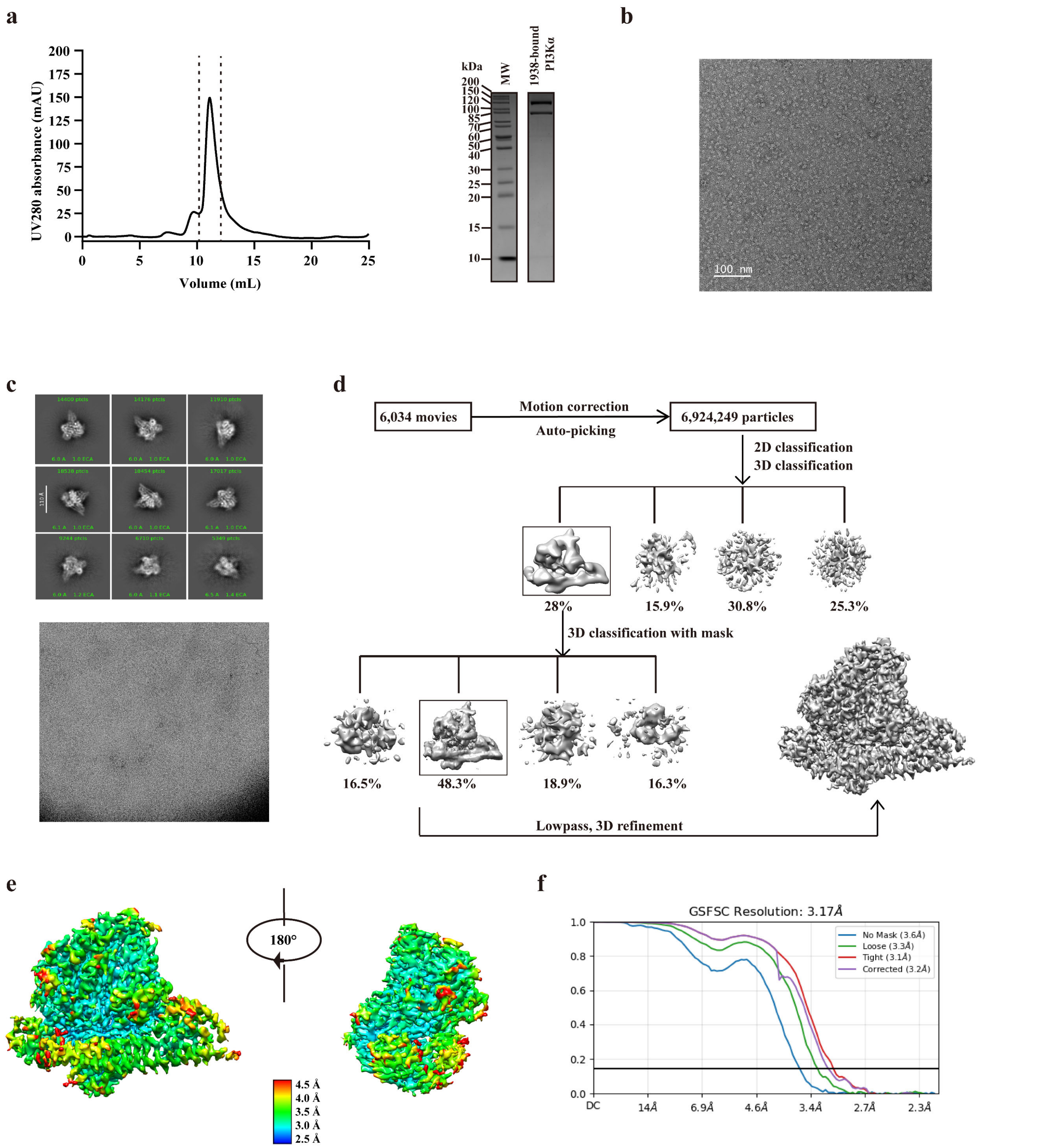

Supplementary Fig. S1 Characterization and cryo-EM analysis of the 1938-bound PI3Kα complex. a Analytical size exclusion chromatography (left) and SDS-PAGE image of the purified PI3Kα complex (right). **b** Negative stain image of the 1938-bound PI3Kα complex. **c** Representative 2D class averages of the 1938-bound PI3Kα complex (top). Representative cryo-EM micrographs of the 1938-bound PI3Kα complex (scale bar: 50 nm) (bottom). **d** Workflow of cryo-EM data processing for the 1938-bound PI3Kα complex. **e** Cryo-EM map of the 1938-bound PI3Kα complex, colored by local resolution (Å). **f** FSC curve of the 1938-bound PI3Kα complex, indicating the resolution is 3.17 Å at a FSC of 0.143.

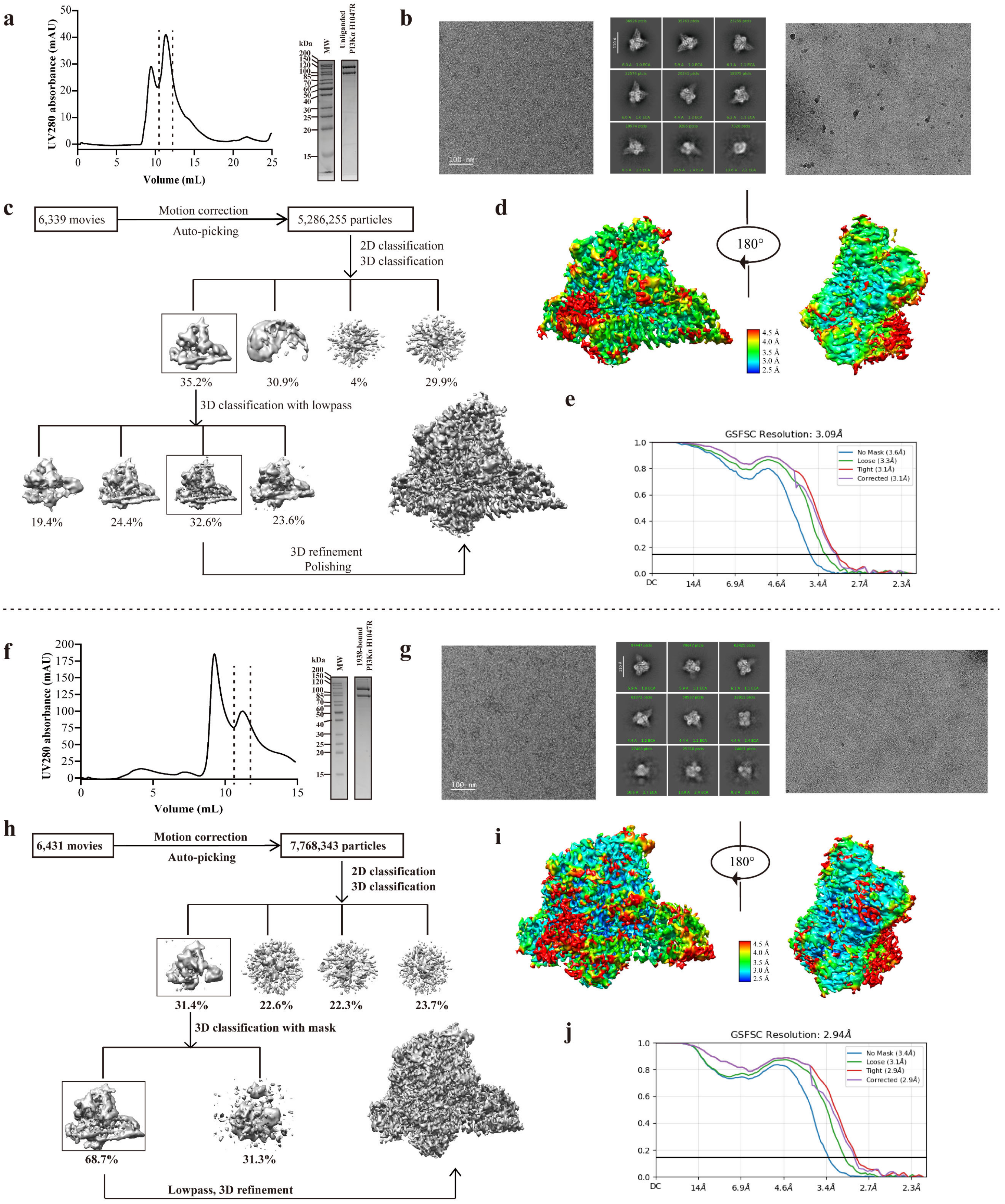

Supplementary Fig. S2 Characterization and cryo-EM analysis of the unliganded H1047R complex and 1938-bound H1047R complex. a Analytical size exclusion chromatography (left) and SDS-PAGE image (right) of the purified H1047R complex. **b** Negative stain image (left) of the H1047R complex. Representative 2D class averages (middle) of the H1047R complex. Representative cryo-EM micrographs (right) of the H1047R complex (scale bar: 50 nm). **c** Workflow of cryo-EM data processing for the H1047R complex. **d** Cryo-EM map of the H1047R complex,

colored by local resolution (Å). **e** FSC curve of the H1047R complex, indicating the resolution is 3.09 Å at a FSC of 0.143. **f** Analytical size exclusion chromatography (left) and SDS-PAGE image (right) of the purified 1938-bound H1047R complex. **g** Negative stain image (left) of the 1938-bound H1047R complex. Representative 2D class averages (middle) of the 1938-H1047R complex. Representative cryo-EM micrographs (right) of the 1938-bound H1047R complex (scale bar: 50 nm). **h** Workflow of cryo-EM data processing for the 1938-bound H1047R complex. **i** Cryo-EM map of the 1938-bound H1047R complex, colored by local resolution (Å). **j** FSC curve of the 1938-bound H1047R complex, indicating the resolution is 3.09 Å at a FSC of 0.143.

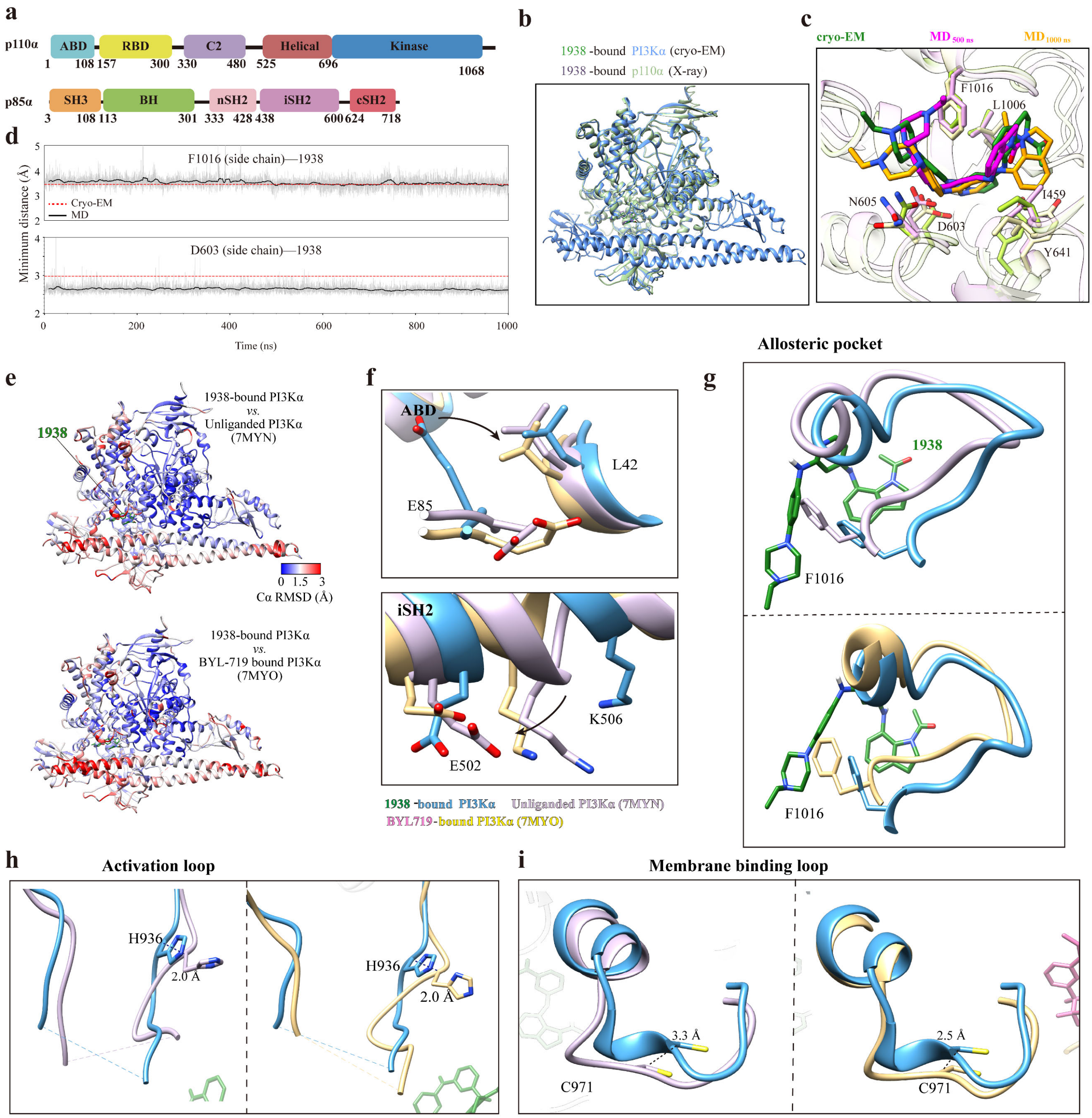

Supplementary Fig. S3 Conformational comparison among 1938-bound, unliganded, and BYL-719-bound PI3Kα structures. **a** Domain structures of PI3Kα. **b** Structural alignment of the cryo-EM structure of the 1938-bound PI3Kα complex (colored blue) and the 1938-bound p110α overall structures (depicted in light green, consistent throughout). **c** Comparison of the binding pose of 1938 in the cryo-EM structure and representative molecular dynamics (MD) simulations snapshots. **d** Representative minimum distances between the non-hydrogen atoms of 1938 and the sidechains of F1016 (top) and D603 (bottom). The thick and thin traces represent moving averages and original, unsmoothed values, respectively. **e** Root-mean-square deviation (RMSD) of Cα atoms between the cryo-EM models of the 1938-bound PI3Kα and unliganded PI3Kα (PDB ID: 7MYN) (top) or BYL-719-bound PI3Kα (PDB ID: 7MYO) (bottom). **f** Representative residue-level conformational changes in the ABD (top) and iSH2 (bottom) in 1938-bound

PI3Kα compared to unliganded and BYL-719-bound states. **g** One loop (residues 1002–1016) shifts from the helical domain towards the active site. **h** The activation loop (residues 933–958) undergoes conformational changes. **i** The membrane-binding loop (residues 966–974) also shows conformational alterations.

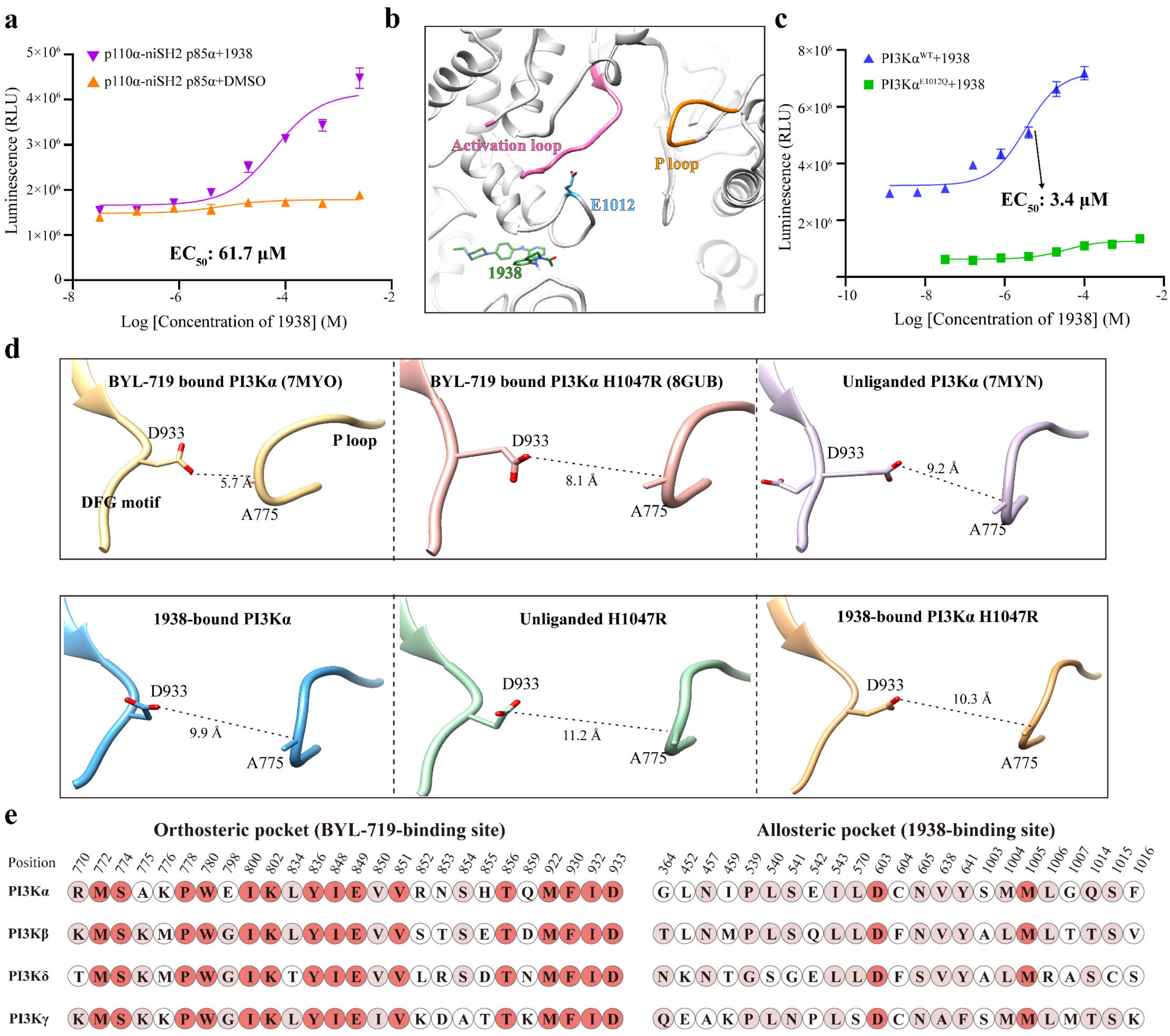

Supplementary Fig. S4. Functional and structural analysis of the 1938-bound WT PI3Kα. a Compound 1938 enhances the kinase activity of a truncated PI3Kα complex comprising p110α and the nSH2 and iSH2 domains of p85α (lacking nSH3, BH, and cSH2 domains), although with reduced potency (EC_{50} = 61.7 μM). **b** Residue E1012 is located in the C-lobe of the kinase domain adjacent to the activation loop and compound 1938. **c** The E1012Q mutation abolished 1938-induced enzymatic activity. **d** Comparison of sidechain positions for A775 and D933 in multiple PI3Kα structures. The minimum distance between the Cα atom of A775 in the P loop and the sidechain oxygen atoms of D933 in the activation loop is shown for each complex, serving as an indicator of pocket openness. **e** Multiple sequence alignment of orthosteric and allosteric pocket residues across PI3K isoforms. Residues within 5 Å of the bound ligands are highlighted to illustrate sequence conservation and variability among isoforms.

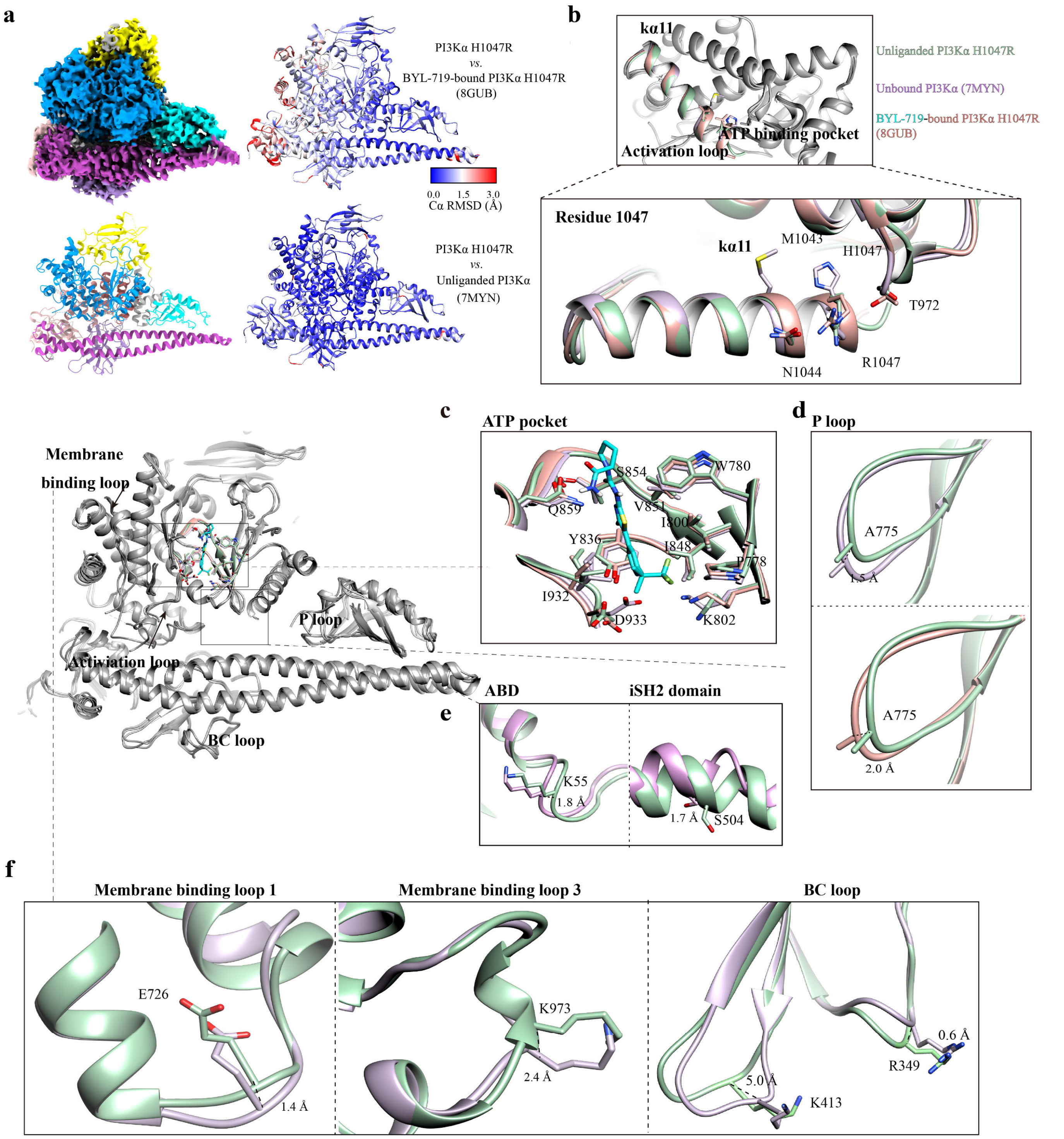

Supplementary Fig. S5 Comparison of the ligand binding mode between PI3Kα H1047R and other ligand-bound PI3Kα structures. **a** Cryo-EM density map (top) and corresponding atomic model (bottom) for the unliganded PI3Kα H1047R complex, shown in two orientations. Cα RMSD values between the cryo-EM models of unliganded and BYL-719-bound PI3Kα H1047R (PDB ID: 8GUB) (top), and unliganded PI3Kα H1047R and WT PI3Kα (PDB ID: 7MYN) (bottom). **b** The conformation of residue H/R1047 in the unliganded PI3Kα H1047R, BYL-719-bound PI3Kα H1047R (PDB ID: 8GUB), and unliganded WT PI3Kα (PDB ID: 7MYN). **c** Residues surrounding the ATP-binding pocket show almost no significant differences among structures, from global and local perspectives. **d** P loop (residues 771–777) differs from those in 8GUB and 7MYN, shifting to position farther from the ATP pocket. A displacement measured of

the Cα carbon of C971 is 3.8 Å. **e** At the interface interacting with p85α, the ABD of p110α has rotated, especially K55 of ABD and S504 of iSH2 domain. **f** The membrane binding loops 1 and 3, as well as the BC loop, show different conformations across the unliganded PI3Kα H1047R, BYL-719-bound PI3Kα H1047R (PDB ID: 8GUB), and unliganded WT PI3Kα (PDB ID: 7MYN) structures.

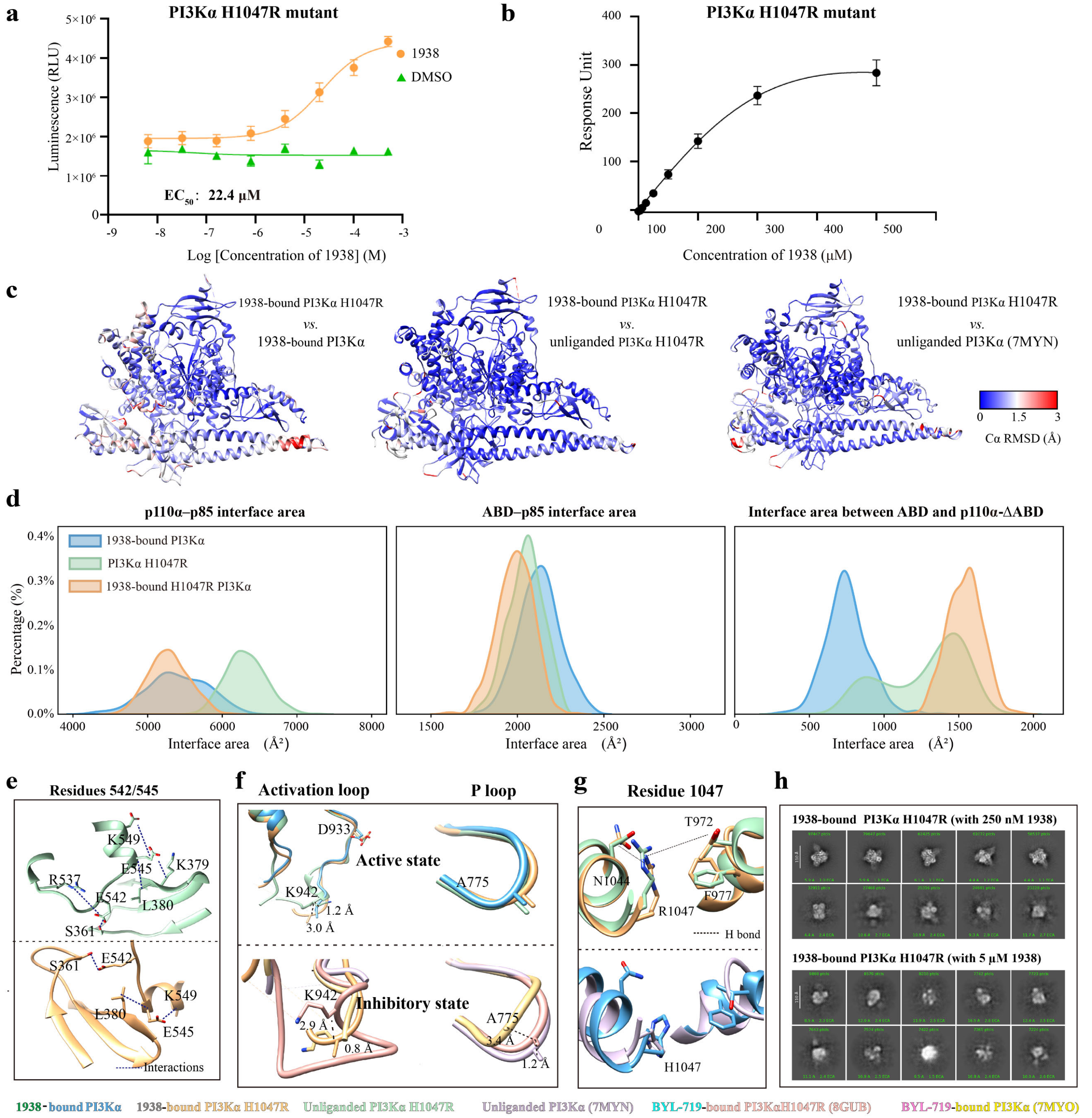

Supplementary Fig. S6 Structural insights into the 1938-bound PI3Kα H1047R complex. a Compound 1938 dose-dependently activates PI3Kα H1047R mutant with an EC_{50} value of 22.4 μM. **b** Representative sensorgram of SPR showing 1938 binding to PI3Kα H1047R (K_D = 261 ± 45 μM). **c** Cα RMSD between the cryo-EM models of the 1938-bound PI3Kα H1047R and 1938-bound PI3Kα WT (left), unliganded PI3Kα H1047R (middle), and unliganded WT PI3Kα (right). **d** Distribution of the interface area between p110α and p85α (left), between ABD and p85α (middle), and between ABD and the p110α without ABD (right), from the last 500 ns of MD simulation trajectories, calculated by FreeSASA 2.0. **e** Residues E542 and E545 show reduced interactions with other residues compared to the H1047R structure. **f** The activation loops of multiple PI3Kα structures (1938-bound PI3Kα, 1938-bound PI3Kα H1047R,

unliganded PI3Kα H1047R, unliganded PI3Kα, BYL-719-bound PI3Kα, BYL-719-bound PI3Kα H1047R) exhibit distinct conformations (left) in active and inhibitory states, respectively, and the P loop of different structures also shows different conformations (right). **g** The conformation of 1047 is consistent with that in the H1047R structure (top), which position is different from 7MYN (bottom). **h** Different concentrations of 1938 (250 nM and 5 μM) display different 2D classifications.

Supplementary Table S1. Cryo-EM data acquisition and image processing.

**Data collection**			
Microscope	Titan Krios		
Voltage (kV)	300		
Corrected Magnification	46,685		
Pixel size (Å)	1.071		
Detector	Gatan K3 Summit		
Defocus range (μm)	−1.5 to −2.5		
Defocus mean (μm)	−2.0		
Total electron exposure ($e^{-}Å^{-2}s^{-1}$)	70		
Exposure rate ($e^{-}Å^{-2}frame^{-1}$)	25		
**Data Processing**	1938-bound PI3Kα	Unliganded PI3Kα H1047R	1938-bound PI3Kα H1047R
Useable image movies	6,034	6,339	6,431
Particles	6,924,249	5,286,255	7,768,343
Final particles	226,201	440,046	1,148,844
Map sharpening B-Factor ($Å^2$)	−123.70	−132.50	−137.10
Resolution (Å)	3.17	3.30	2.94
Unmasked (0.143 FSC)	3.60	3.60	3.40
Masked (0.143 FSC)	3.20	3.10	2.90
Local resolution range (Å)	2.90−10.60	2.75−11.31	2.56−10.64
**Model**			
EMDB accession code	EMD-63456	EMD-63457	EMD-63458
PDB accession code	9LWQ	9LWR	9LWS
Model Composition			
Chains	3	2	2
Non-hydrogen atoms	9842	10,398	9,848
Protein Residues	1206	1,261	1215
Refinement			
Software	phenix.real_space_refine	phenix.real_space_refine	phenix.real_space_refine
Resolution (Å)	3.17	3.09	2.94
CC (mask)	0.73	0.67	0.60
CC (peaks)	0.61	0.57	0.50
CC (volume)	0.70	0.65	0.57
Average B Factor ($Å^2$)			
Protein	46.91	68.59	39.40
Ligand	15.33	--	--

R.M.S. deviations			
Bond Length (Å) (# > 4σ)	0.003 (0)	0.003 (0)	0.004 (0)
Bond Angles (°) (# > 4σ)	0.449 (1)	0.569 (0)	0.653 (2)
Validation			
MolProbity Score	2.52	1.92	2.03
Clashscore, all atoms	36.02	11.52	13.96
Rotamer outliers (%)	0.00	0.00	0.09
Cβ outliers (%)	0.00	0.00	0.00
CaBLAM outliers (%)	3.01	3.35	2.71
Ramachandran plot (%)			
Outliers	0.00	0.00	0.00
Allowed	7.77	4.98	5.51
Favored	92.23	95.02	94.40

References

1. Liu X, Yang S, Hart JR *et al.* Cryo-EM structures of PI3Kalpha reveal conformational changes during inhibition and activation. *Proc Natl Acad Sci U S A* 2021; **118**:e2109327118.

2. Liu X, Zhou Q, Hart JR *et al.* Cryo-EM structures of cancer-specific helical and kinase domain mutations of PI3Kalpha. *Proc Natl Acad Sci U S A* 2022; **119**:e2215621119.

3. Hart JR, Liu X, Pan C *et al.* Nanobodies and chemical cross-links advance the structural and functional analysis of PI3Kalpha. *Proc Natl Acad Sci U S A* 2022; **119**:e2210769119.

4. Zhou Q, Liu X, Neri D *et al.* Structural insights into the interaction of three Y-shaped ligands with PI3Kalpha. *Proc Natl Acad Sci U S A* 2023; **120**:e2304071120.

5. Zheng SQ, Palovcak E, Armache JP, Verba KA, Cheng Y, Agard DA. MotionCor2: anisotropic correction of beam-induced motion for improved cryo-electron microscopy. *Nat Methods* 2017; **14**:331-332.

6. Zhang K. Gctf: real-time CTF determination and correction. *J Struct Biol* 2016; **193**:1-12.

7. Moriarty NW, Grosse-Kunstleve RW, Adams PD. electronic Ligand Builder and Optimization Workbench (eLBOW): a tool for ligand coordinate and restraint generation. *Acta Crystallogr D Biol Crystallogr* 2009; **65**:1074-1080.

8. Terwilliger TC, Klei H, Adams PD, Moriarty NW, Cohn JD. Automated ligand fitting by core-fragment fitting and extension into density. *Acta Crystallogr D Biol Crystallogr* 2006; **62**:915-922.

9. Pettersen EF, Goddard TD, Huang CC *et al.* UCSF Chimera--a visualization system for exploratory research and analysis. *J Comput Chem* 2004; **25**:1605-1612.

10. Pettersen EF, Goddard TD, Huang CC *et al.* UCSF ChimeraX: structure visualization for researchers, educators, and developers. *Protein Sci* 2021; **30**:70-82.

11. Abraham MJ, Murtola T, Schulz R *et al.* GROMACS: High performance molecular simulations through multi-level parallelism from laptops to supercomputers. *SoftwareX* 2015; **1-2**:19-25.

12. Sastry GM, Adzhigirey M, Day T, Annabhimoju R, Sherman W. Protein and ligand preparation: parameters, protocols, and influence on virtual screening enrichments. *J Comput Aided Mol Des* 2013; **27**:221-234.

13. Lee J, Cheng X, Swails JM *et al.* CHARMM-GUI input generator for NAMD, GROMACS, AMBER, OpenMM, and CHARMM/OpenMM simulations uing the CHARMM36 additive force field. *J Chem Theory Comput* 2016; **12**:405-413.

14. Huang J, Rauscher S, Nawrocki G *et al.* CHARMM36m: an improved force field for folded and intrinsically disordered proteins. *Nat Methods* 2017; **14**:71-73.

15. Vanommeslaeghe K, Hatcher E, Acharya C *et al.* CHARMM general force field: a force field for drug-like molecules compatible with the CHARMM all-atom additive biological force fields. *J Comput Chem* 2010; **31**:671-690.

16. Hess B. P-LINCS: A Parallel Linear Constraint Solver for Molecular Simulation. *J Chem Theory Comput* 2008; **4**:116-122.

17. Aoki KM, Yonezawa F. Constant-pressure molecular-dynamics simulations of the crystal-smectic transition in systems of soft parallel spherocylinders. *Phys Rev A* 1992; **46**:6541-6549.

18. Mitternacht S. FreeSASA: an open source C library for solvent accessible surface area calculations. *F1000Res* 2016; **5**:189.
